# Family influences on older adults' problem drinking: A representative nationwide study of China

**DOI:** 10.3389/fpubh.2022.850931

**Published:** 2022-08-02

**Authors:** Yaping Ye, Jian Feng, Yeyuan Zhang, Manli Wang, Jinsong Chen, Dan Wu, Young Kathleen, Shuhan Jiang

**Affiliations:** ^1^School of Humanities and Management, Zhejiang Chinese Medical University, Hangzhou, China; ^2^National Institute for Health Innovation, School of Population Health, University of Auckland, Auckland, New Zealand; ^3^School of Psychology, Center for Mental Health, Shenzhen University, Shenzhen, China; ^4^Young Kathleen, Department of Health Sciences, MPH and Public Health Education Programs, California State University, Los Angeles, CA, United States

**Keywords:** aging, problem drinking, family factors, alcohol consumption, the elderly

## Abstract

**Aim:**

It is reported that problem drinking is severe among the elderly. The family environment has been regarded as a significant effecting factor in alcohol consumption of the drinker. With the increasing number of older people, paying more attention to this vulnerable group's drinking status and its' influencing factors is substantial for improving older adults' health and the quality of health services.

**Methods:**

This study used data from the Chinese Longitudinal Healthy and Longevity Study (CLHLS), which was a representative survey covering 23 provinces in mainland China. Cross-sectional analyses were conducted with 15,142 older individuals (aged ≥65 years). Three self-reported questions about drinking behavior were examined to calculate alcohol consumption and categorize problem drinkers. Three multi-level models were utilized while adjusting for numerous socio-demographic and self-reported health factors to analyze the effect of family factors associated with problem drinking among the elderly.

**Results:**

A total of 1,800 problem drinkers (12%) were identified in the sample. Key factors for the problem drinker were assessed such as Hukou (governmental household registration system), current marital status, years of schooling, primary caregivers, and financial sources of living were associated with problem drinking. The older population who live in rural areas (OR = 1.702, CI = 1.453, 1.994), with advanced years of education (OR = 1.496, CI = 1.284, 1.744), and making life by themselves (OR = 1.330, CI = 1.139, 1.552) were more likely to engage in problem drinking while those participants who are widowed (OR = 0.678, CI = 0.574, 0.801), cared for by children or other relatives (OR = 0.748, CI = 0.642, 0.871), adult care giver (OR = 0.348, CI = 0.209, 0.578) or by no one (OR = 0.539, CI = 0.348, 0.835), provided with financial support from their children (OR = 0.698, CI = 0.605, 0.806), other relatives (OR = 0.442, CI = 0.332, 0.587), or the government/community (OR = 0.771, CI = 0.650, 0.915), with insufficient financial support (OR = 0.728, CI = 0.608, 0.872) were at lower risk of problem drinking.

**Conclusions:**

This study provides a strong correlation of various family factors that were associated with problem drinking among the elderly. The findings underscore the effort to promote healthy behaviors, including the importance of positive family factors and appropriate levels of alcohol consumption.

## Introduction

It's widely known that the harmful use of alcohol is one of the leading risk factors for population health worldwide across all age groups (1). But alcohol abuse and alcoholism are neglected among older adults (2). Data from the National Survey on Drug Use and Health showed that nearly 11% of older adults over age 65 reported current binge drinking (3). As the World Health Organization (WHO) has shown, alcohol consumption affects the risks of almost 230 three-digit diseases and injury codes in the International Statistical Classification of Diseases and Related Health Problems: 10th Revision (ICD-10) (1). Such as injury, alcoholic liver disease, heart disease, stroke, cancer, and Gastrointestinal disease (4), which may cause premature death. Many high-income countries seem to incur 1% or more of their gross domestic product (GDP) as alcohol-attributable costs each year, placing an enormous burden on public health (5).

Due to the slower alcohol metabolism in older adults, the effects of alcohol consumption are likely prolonged and potentially worsened (6–8). However, many older people are skeptical about the health risks of drinking (9), and risky drinking is increasing among older people (10). It is a well-known that aging occurs in the body with increasing age, which causes different degrees of immunity decline, hypohepatia, and other problems. That is why drinking alcohol is even more likely to be harmful to people aged 65 and above (11). Intoxication may also increase the risk of falls in older adults, which could be fatal (12). Thus, further research needs to examine the potential risk factors associated with alcohol use in order to provide preventive measures for senior people at an earlier age, which may improve their health status and reduce long-term health loss.

Meanwhile, population aging challenges both developed and developing countries (13). According to the latest data released by the United States Bureau of the Census (USBC), there are 16.5% of Americans over 65 years old. The elderly population in the United States is projected to rise to 74 million by 2030 (14). As for China, in recent decades, the level of public health services and medical treatment has been significantly improved, which has contributed to a large increase in the average life expectancy. The seventh national census in 2020 showed the number of people aged 60+ on the mainland reached 264 million, accounting for 18.7% of the total population (15). In this context, the health problems of the elderly should raise particular concerns.

Existing research concerning the family environment has been regarded as a significant factor in alcohol consumption of adolescents and middle-aged people (16, 17). According to Rucker et al., the parent is a determining cause of their children's use of alcohol (18). In contrast, others applied the theory of “role incompatibility” to explain the decrease in intoxication and more problem drinking after their marriage (16). Previous studies have shown that family factors play an essential role in the pathogenesis, treatment, and recovery of substance abuse use of patients (19). Yet, few studies on the causes of elderly drinking at the family level have been examined. Therefore, the authors of this study, sought to assess the potential association between family factors and problem drinking in an extensive Chinese representative nationwide data of older adults.

## Method

### Data source

In this study, we used the data from the Chinese Longitudinal Healthy Longevity Survey (CLHLS), the first and largest nationwide community-based, longitudinal prospective cohort survey concerning older adults in China (20). It randomly selected approximately one-half of the counties of the 23 provinces as primary survey units. To understand the health status and associated social, behavioral, and biological factors among Chinese older people, the CLHLS conducted a baseline survey in 1998 and followed up almost every 2 years, 2002, 2005, 2008, 2011, 2014, and 2018. We used the most recent cross-sectional data (2018) of the CLHLS which provided information regarding demographic characteristics variables, socioeconomic status, and social support variables of Chinese older adults. The research team employed this survey to explore the potential correctional effects of the family environment and problem drinking. After filtering for missing values, outliers, etc., our analysis included 15,142 older people aged 65+, including 6,634 males and 8,508 females.

### Data collection

Individual variables involved in this study were measured *via* a self-reported questionnaire conducted by health professionals from the Center for Healthy Aging and Development at Peking University. The questionnaire was finished face-to-face by the participants themselves or with the assistance of their family members. All responses were anonymous and confidential. The files are secured in a locked filing cabinet and accessible only to the researchers of this study. The data were input by professionals to ensure integrity and accuracy.

### Measures

#### Dependent variable

WHO once defined “problem drinking” as a pattern of drinking that increases drinkers' risk of (potentially) harmful consequence. According to a combined analysis of individual participant data of 599,912 current drinkers in 83 prospective studies with the level of alcohol consumption over 100 g per week, a positive association was recorded with all-cause mortality (21). Based on the above findings, this paper defines “problem drinking” by three questions: 1. Are you drinking currently? 2. What kind of wine do you mainly drink? 3. How much alcohol do you drink per day? The research team converted the data collected for this study on alcohol consumption by type and amount of alcohol consumed. Fifty ml of high proof-alcohol liquor is equal to ~25 g of alcohol. Fifty ml of low proof -alcohol liquor (sorghum liquor, medicinal liquor) is equal to 16.667 g of alcohol. Fifty ml of rice wine or fruit wine is equal to ~4 g alcohol, 50 ml of beer is equal to ~1.67 g of alcohol and 50 ml of yellow rice wine is equal to ~6 g of alcohol. Accordingly, drinking more than 100 grams of pure alcohol per week is categorized as “problem drinking”.

#### Independent variables

Considering the context of “problem drinking” among the elderly our study selected 13 independent variables: Hukou (community, the place of registered permanent residence), age, ethnicity, current marital status, year of schooling, occupation before 60-year-old, self-reported economic status, self-reported health status, people they cohabited with, primary caregivers, financial status and level of sufficient stature of living condition.

The age in the questionnaire was calculated by the participants birth year and respondents 65+ were tabulated for the study, and the data were divided into three categories: 65–80 years old, 80–95 years old, and over 95 years old. Ethnicity in the questionnaire was categorized by two categories: Han or, non-Han minority.

The questionnaire asked the respondents to self-report their health status with the question “How do you feel about your health status now?” using the ranking of good, average, poor, no response).

Marriage was considered a source of social support and has been shown as a protective factor of the problem drinker's level of use and abuse (22). Additional marital status questions were included such as: “what's your current marriage status?” Marital status was recorded into four categories: Married and living together, married without living together, divorced, widowed and never married.

Studies also indicated that an individual's educational level is another risk factor for problem drinking (23). The research team applied the following categories: 0, 1–6, and 6 years and above. In 1966, the Cultural Revolution broke out in China, the education system was destroyed, and it hadn't been rebuilt from the ruins until the Compulsory Education Law was enacted in 1986 which required every child to complete 9 years of formal schooling (6 years for primary and 3 years for junior and secondary schooling) (24). As a result, most people were less educated at that time and people who received education for no <6 years are regarded as educated now.

Occupation was recorded in 8 categories: Professional and technical personnel, commercial, service. In addition, operational workers were categorized as agricultural, forestry, animal husbandry or fishery worker. Additional categories were provided: Self-employed, house-worker, military personnel, never worked and other. Since income status may influence drinking patterns (25), participants' economic statuses were assessed. The options included: Rich, Average, and Poor. We also asked who offered the financial sources of living to them and whether it was sufficient or not.

Several theoretical perspectives, including The Role Incompatibility Theory (26), Routine Activity Theory (27), and Age-graded Social Control Theory (28) posit that women and men reduce alcohol consumption when they transition into adult roles because heavy drinking conflicts with various levels of success in adulthood. Therefore, we raised a presumption that the family roles of the elderly are associated with problem drinking.

Questions about cohabitation status such as “Who are you currently living with?” Included responses such as with a family member, living alone, and living in a nursing home. Caregiving support questions included: “Currently, who is the main caregiver when you are unwell or sick?” Responses included a spouse, with children and/ or other relatives, neighbors, social workers, baby-sitter or, no one.

### Data analysis

All survey data were entered into a Microsoft Excel database. SPSS version 23.0 for windows was used for the data analyses. Descriptive statistics were calculated to determine the prevalence of problem drinking. The differences in problem drinking among old adults across sociodemographic characteristics were analyzed using the Chi-square test. The independent variables in the logistic regression analysis were those variables that emerged as statistically significant for problem drinking in the Chi-square tests. A Wald test was used to test the statistical significance of each coefficient in the model. The odds ratio (OR) expresses the relative likelihood of being a problem drinker. ORs and 95% CIs are reported. The significance level was set at *p* < 0.05. We also constructed three models for logistic regression analyses of problem drinking to the role of individual behavior and family-related factors. We started with the Null Model. Model 1 was the individual model, which included statistically significant sociodemographic variables and individual behavior factors. Model 2 was the family model, which only included family-related factors. In the combined model (Model 3), all factors, including statistically significant sociodemographic variables and individual and family factors, were examined simultaneously.

## Result

### Sociodemographic characteristics

Fifteen thousand one hundred forty-two older adults completed the questionnaire. Table 1 showed the various characteristics of the participants. The result showed that there were 1,800 problem alcohol users in this sample and the prevalence of problem alcohol users among the elderly for the urban and rural samples was 10.9% (weighted) and 12.3%, respectively. The prevalence of problem alcohol users for the elderly who had never married was 18.8% while the widowed was 8.4%. As for the education experience, the prevalence of problem alcohol users for those who had never accepted education was 8.6%. The ratio of problem drinkers to those who had gotten an education for more than 6 years was 16%. Other sociodemographic characteristics of the study participants were also shown in the Table 1.

**Table 1:** Sociodemographic characteristics of the study sample (*N* = 15,142).

**Variable**	***N***	**% of total**	**Problem alcohol users (*n*)**	**Problem alcohol users' prevalence**	**χ^2^ (*P*)**
**Hukou**					5.79, *P* = 0.016
Urban	4,188	27.7	455	10.9	
Rural	10,954	72.3	1,345	12.3	
**Sex**					0.69, *P* = 0.418
Male	6,634	43.8	805	12.1	
Female	8,508	56.2	995	11.7	
**Age**					158.76, *P <* 0.01
65–80	5,133	33.9	835	16.3	
80–95	5,936	39.2	637	10.7	
≥95	4,073	26.9	328	8.1	
**Ethnicity**					4.80, *P* = 0.030
Han	14,153	93.5	1,704	12.0	
Minority	989	6.5	96	9.7	
**Current marital status**					235.27, *P <* 0.01
Married and living together	5,902	39.0	976	16.5	
Married without living together/divorced	356	2.4	60	16.9	
Widowed	8,751	57.8	739	8.4	
Never married	133	0.9	25	18.8	
**Year of schooling**					153.49, *P <* 0.01
0	7,326	48.4	631	8.6	
1–6	3,985	26.3	555	13.9	
>6	3,831	25.3	614	16.0	
**Occupation before 60-year-old**					41.37, *P <* 0.01
Professional and technical personnel	871	5.8	128	14.7	
Governmental, institutional, or managerial personnel	543	3.6	94	17.3	
Commercial, service, and operational workers	2,074	13.7	256	12.3	
agriculture, forestry, animal husbandry, or fishery worker	9,643	63.7	1,113	11.5	
Self-employed or house-worker	1,425	9.4	132	9.3	
Military personnel	136	0.9	17	12.5	
Never worked	214	1.4	19	8.9	
Other	236	1.6	41	17.4	
**Self-report economic status**					9.85, *P* <0.01
Rich	2,889	19.1	379	13.1	
Average	10,602	70.0	1,256	11.8	
Poor	1651	10.9	165	10.0	
**Self-reported health status**					4.32, *P* < 0.01
Good	6,523	43.1	805	12.3	
Average	5,381	35.5	641	11.9	
Poor	1,983	13.1	221	11.1	
Could not answer	1,255	8.3	133	10.6	
**People they live with**					14.99, *P* < 0.01
Family member	12,167	80.4	1,471	12.1	
Living alone	2,420	16.0	292	12.1	
In a nursing home	555	3.7	37	6.7	
**Primary caregivers**					237.76, *P <* 0.01
Spouse	4,123	27.2	758	18.4	
Children/other relatives	10,079	66.6	973	9.7	
Neighbors/social workers	277	1.8	25	9.0	
Adult care giver	394	2.6	18	4.6	
Nobody	269	1.8	26	9.7	
**Financial sources of living**					111.48, *P <* 0.01
Self-reliance	5,064	33.4	791	15.6	
Children	6,977	46.1	679	9.7	
Other relatives	779	5.1	60	7.7	
Government/community	2,322	15.3	270	11.6	
**Sufficient or not**					18.13, *P <* 0.01
Yes	12,977	85.7	1,602	12.3	
No	2,165	14.3	198	9.1	

### Influencing factors of problem drinking

The research team utilized three models to investigate the influencing factors of problem drinking: the individual model, family model, and combined model. The results were reported in Table 2. Of the sociodemographic characteristics, living in a rural area (OR = 1.702, CI = 1.453–1.994) and more education were indicated as risk factors for problem drinking. Compared to those who were never educated to those who had schooling for 1–6 years (OR = 1.409, CI = 1.240–1.602) or no <6 years (OR = 1.496, CI = 1.284–1.744) are 1.409 and 1.496 times predisposed to problem drinking, respectively. Widowed elderly is not prone to problem drinking. As for the family level, the elderly who are looked after by children or other relatives or babysitters tends to avoid problem drinking. People who live alone have a higher risk of problem drinking than those living with family members. At the same time, compared to self-reliance, the elderly who get financial sources of living assistance from children (OR = 0.698, CI = 0.605–0.806) or other relatives (OR = 0.442, CI = 0.332–0.587) or government and community (OR = 0.771, CI = 0.650–0.915) are less likely to be associated with problem drinking. Insufficient financial sources (OR = 0.728, CI = 0.608–0.872) are significantly inversely associated with a higher risk of problem drinking.

**Table 2:** Result of multilevel models.

**Group**	**Null model OR (95% C.I.)**	**Model 1 individual model OR (95% C.I.)**	***P***	**Model 2 family model OR (95% C.I.)**	***P***	**Model 3 combined model OR (95% C.I.)**	***P***
**Hukou**							
Urban		1.00				1.00	
Rural		**1.599 (1.374,1.806)**	**<0.001**			**1.702 (1.453,1.994)**	**<0.001**
**Age**							
65–80		1.00				1.00	
80–95		**0.837 (0.740, 0.946)**	**0.004**			0.911 (0.803, 1.034)	0.150
≥95		**0.791 (0.670, 0.933)**	**0.005**			0.933 (0.785, 1.109)	0.432
**Ethnicity**							
Han		1.00				1.00	
Minority		**0.773 (0.620, 0.963)**	**0.022**			0.805 (0.645, 1.006)	0.056
**Current marital status**							
Married, and living together		1.00				1.00	
Married without living together/Divorced		1.108 (0.830, 1.478)	0.488			1.191 (0.879, 1.613)	0.260
Widowed		**0.595 (0.526, 0.673)**	**<0.001**			**0.678 (0.574, 0.801)**	**<0.001**
Never married		1.314 (0.841, 2.055)	0.230			1.513 (0.93, 2.456)	0.094
**Year of schooling**							
0		1.00				1.00	
1–6		1.442 (1.269, 1.637)	**<0.001**			**1.409 (1.240, 1.602)**	**<0.001**
≥6		1.579 (1.357, 1.837)	**<0.001**			**1.496 (1.284, 1.744)**	**<0.001**
**Occupation before 60-year-old**							
Professional and technical personnel		1.00				1.00	
Governmental, institutional or managerial personnel		1.300 (0.968, 1.746)	0.081			1.293 (0.961, 1.740)	0.090
Commercial, service and operational workers		0.950 (0.751, 1.202)	0.669			0.940 (0.741, 1.191)	0.607
agriculture, forestry, animal husbandry, or fishery worker		0.848 (0.666, 1.079)	0.180			0.955 (0.745, 1.223)	0.713
Self-employed or house-worker		**0.724 (0.542, 0.965)**	**0.028**			0.819 (0.611, 1.099)	0.184
Military personnel		0.854 (0.492, 1.482)	0.574			0.892 (0.512, 1.554)	0.687
Never worked		0.922 (0.545, 1.560)	0.762			1.110 (0.651, 1.893)	0.702
Other		1.307 (0.877, 1.948)	0.189			1.395 (0.932, 2.086)	0.105
**Self-reported economic status**							
Rich		1.00				1.00	
Average		0.920 (0.809, 1.045)	0.198			0.933 (0.820, 1.061)	0.291
Poor		**0.778 (0.636, 0.953)**	**0.015**			0.937 (0.746, 1.176)	0.573
**Self-reported health status**							
Good		1.00				1.00	
Average		0.968 (0.865, 1.083)	0.575			0.968 (0.864, 1.083)	0.567
Poor		0.897 (0.764, 1.053)	0.184			0.897 (0.764, 1.054)	0.188
Could not answer		0.854 (0.701, 1.040)	0.117			0.864 (0.708, 1.052)	0.146
**People they live with**							
Family member				1.00		1.00	
Living alone				**1.284 (1.113, 1.482)**	**0.001**	**1.330 (1.139, 1.552)**	**<0.001**
In a nursing home				**0.661 (0.444, 0.982)**	**0.040**	0.777 (0.519, 1.163)	0.220
**Primary caregivers**							
Spouse				1.00		1.00	
Children/other relatives				**0.495 (0.442, 0.555)**	**<0.001**	**0.748 (0.642, 0.871)**	**<0.001**
Neighbors/social workers				**0.553 (0.340, 0.899)**	**0.017**	0.715 (0.430, 1.187)	0.195
Adult care giver				**0.222 (0.136, 0.361)**	**<0.001**	**0.348 (0.209, 0.578)**	**<0.001**
Nobody				**0.445 (0.292, 0.679)**	**<0.001**	**0.539 (0.348, 0.835)**	**0.006**
**Financial sources of living**							
Self-reliance				1.00		1.00	
Children				**0.709 (0.631, 0.798)**	**<0.001**	**0.698 (0.605, 0.806)**	**<0.001**
Other relatives				**0.456 (0.346, 0.601)**	**<0.001**	**0.442 (0.332, 0.587)**	**<0.001**
Government/community				**0.812 (0.697, 0.946)**	**0.007**	**0.771 (0.650, 0.915)**	**0.003**
**Sufficient or not**							
Yes				1.00		1.00	
No				**0.752 (0.642, 0.882)**	**<0.001**	**0.728 (0.608, 0.872)**	**0.001**
Fixed parameters	−2.003^**^	−1.944^**^		−1.301^**^		−1.785^**^	

** *P* < 0.01.

## Discussion

The study results indicated an association between rural-urban dwellers and problem drinking, which is consistent with previous research (29, 30). Older adults living in rural areas are more likely to be problem drinkers than those in the urban district. The community environment is a known determinant of older adults' health and the existing literature has found that individuals who lived in a neighborhood with a poorly developed environment were more likely to report heavy drinking compared with those living in adequate and sufficient environment (31). In the past few decades, China has implemented the construction concept of “emphasizing cities over villages.” The gap between urban and rural residential areas in terms of living and access to environmental public facilities is prevalent. There are problems in rural areas such as low quality of public facilities, low level of security, and a general lack of a healthy living environment (32). In this context, there is also a severe imbalance in the quality, quantity, and availability of public health services between urban and rural areas (33). It is difficult for old adults in rural areas to obtain health education resources that provide and positively impact their health literacy (i.e., the capacity of understanding and accessing health information, making appropriate decisions about health, and translating this knowledge into the ability to live healthfully on a daily basis) (34). Thus, older adults in rural areas were less likely to recognize the dangers of drinking, leading to problem drinking.

Unlike previous studies, our study indicated that widowed older adults have a lower likelihood of problem drinking than those married and living with their spouses. Although some studies suggested that the population who are widowed has a higher chance of depression and a higher propensity for concurrent substance use disorders (35, 36). Some studies have suggested the opposite: widowhood does not always have a negative impact (37, 38). Since older adults regularly suffered from multiple diseases and often were very ill before passing away, the physical and mental burdens associated with caring for a sick spouse can compromise the health of caregivers (37). So, being widowhood, to some extent, would make them aware of the importance of having time to themselves and improve the health of caregivers (38). Due to having more free time, studies have found that being widowhood is associated with the adoption of healthy behaviors such as increased likelihood of physical activity (39, 40). Thus, the association between current marital status and problem drinking may possibly occur from these likely mechanisms: “a decline in mental health beginning in the first 2 years before spousal death, lasting up to 2 years after spousal death” (41), and the health and mental health status of the widowed elderly population is different based on the different stages of grief (42). After the initial shock by spousal bereavement, the widowed should be aware of the importance of engaging in healthy behaviors in order to regain a healthy lifestyle (39, 40). Secondly, the social effects and occasions of alcohol consumption from the spouse of widowed elderly are less than those of married and couples living-together. This information attests that the interdependence between spouses was an important factor influencing the drinking patterns (43, 44).

Recent studies have found that older adults with more years of education have a higher likelihood of problem drinking than the uneducated in a Chinese sample population. Advanced education increasingly leads to better promotional opportunities in one's career and to reach a higher socioeconomic status (SES). It was widely convinced that individuals with a higher SES are more likely to consume alcohol compared with individuals with a lower SES (45–48). Higher SES provides individuals with accessible income for their drinking habits. Secondly, higher SES always means higher social capitals (49, 50) which are based on social participation and social networks (51, 52). Alcohol is used as a medium for emotional communication, especially among men (46). Furthermore, most of the older adults in this sample were born and raised in an age of material deprivation. Alcohol is generally considered a rare commodity. For older adults with more education, according to Behavioral Accessibility Theory, greater engagement in social activities may foster greater alcohol availability, in turn increasing one's susceptibility to problem drinking (53–55).

Our study also found that compared with self-reliance, the study participants who got financial sources from children, other relatives, government and/or the community are associated with less problem drinking. Conversely, with the development of a social security system, older people could potentially live on a stable pension (56). Additionally, the transformation of China's industrial structure has provided a number of suitable service jobs for the elderly thereby increasing the labor force's participatory rate (57). Opportunities for wage increases provide the elderly populace with stable sources of income in their later years. Specifically, self-reliant seniors have higher economic dominance to pay for personal consumption (58), including alcohol. As for the elderly supported by children or other relatives, they may be offered very limited financial support from their children and relatives referring to the Generational Imbalance Theory (58). This is a phenomenon that does not conform to Chinese filial piety (59), but surprisingly creates a protective mechanism to prevent the elderly from problem drinking. Another source of financial support is generally subsidies and relief funds from the government or community. The amount of which is too small to cover the cost of alcohol. A similar conclusion was reached by previous research when the discretionary income of the elderly decreases, excessive alcohol consumption might be suppressed (60). This also explains another result in the present study that older adults are less likely to have problem drinking when their sources of living are insufficient. These findings did suggest that family environment is strongly associated with drinking among the elderly.

## Conclusion

Most of the existing studies about the influential factors of problem drinking are youth and young adult populations. Our study focused on the elderly population. The investigators of this study attempted to explain the phenomenon and the mechanism of how family factors affect problem drinking among the elderly. This study indicates that health policies must also pay attention to the drinking problem among older adults. Considering the influencing family factors of problem drinking, health officials should devise more targeted solutions to improve elderly health.

## Limitations

The present study has several limitations. First, our study used one cross-sectional dataset, so no causal inferences could be drawn about family factors and problem drinking. However, the old population behavior pattern observed by such a nationally representative sample would provide valuable information for health promotion programs. Second, the single-resided elder is exploding in China caused of the trend of aging. Although whether older people live alone or not was involved in this study as an essential dependent variable, we recommend future review. Third, the data of alcohol consumption was based on self-report instead of biological measurement. Even though older people with severe impairment of memory or cognitive function would be excluded for the data quality control, problem-drinking behavior may be under or overestimated because of reporting bias.

## Data availability statement

Publicly available datasets were analyzed in this study. This data can be found here: http://opendata.pku.edu.cn.

## Ethics statement

This study used data from the Chinese Longitudinal Healthy and Longevity Study (CLHLS, 2018). The CLHLS study was approved by the research Ethics Committees of Duke University and Peking University (IRB00001052–13074). Informed consent was obtained from all participants before the study and by including a consent statement in each survey round as the initial question, whereby participants needed to agree to progress. All methods were carried out by relevant guidelines and regulations.

## Author contributions

SJ designed the study and analyzed the data. YY, JF, YZ, and MW interpreted the data. YY and SJ drafted the manuscript. JC, DW, and YK revised the manuscript. All authors contributed to the article and approved the submitted version.

## Funding

This study was partially funded by the Nature Science Foundation of Zhejiang Province (LQ20G030014), 2020 Educational Science Planning Project of Zhejiang Province (2020SCG367), and 2021 Zhejiang University Student Science and Technology Innovation Activity Plan (Xinmiao Talent Plan) (2021R410064).

## Conflict of interest

The authors declare that the research was conducted in the absence of any commercial or financial relationships that could be construed as a potential conflict of interest.

## Publisher's note

All claims expressed in this article are solely those of the authors and do not necessarily represent those of their affiliated organizations, or those of the publisher, the editors and the reviewers. Any product that may be evaluated in this article, or claim that may be made by its manufacturer, is not guaranteed or endorsed by the publisher.
